# New Systemic Inflammatory Indices as Predictors of Fulminant Myocarditis in Children

**DOI:** 10.3390/diagnostics15080961

**Published:** 2025-04-10

**Authors:** Demet Kangel, İsa Ozyılmaz, Sercin Ozkok, Hatice Dilek Özcanoğlu, Ali Nazım Güzelbağ, Burcu Çevlik, İbrahim Cansaran Tanıdır, Ali Can Hatemi, Erkut Öztürk

**Affiliations:** 1 Division of Pediatric Cardiology, Department of Pediatrics, Basaksehir Cam and Sakura City Hospital, Istanbul 34480, Turkey; isaozyilmaz@gmail.com (İ.O.); anguzelbag@gmail.com (A.N.G.); drburcudrl@gmail.com (B.Ç.); cansaran@yahoo.com (İ.C.T.); erkut_ozturk@yahoo.com (E.Ö.); 2 Department of Radiology, Basaksehir Cam and Sakura City Hospital, Istanbul 34480, Turkey; sercinbas2005@gmail.com; 3 Department of Anesthesiology and Reanimation, Basaksehir Cam and Sakura City Hospital, Istanbul 34480, Turkey; dilekmersin@hotmail.com; 4 Department of Pediatric Cardiovascular Surgery, Basaksehir Cam and Sakura City Hospital, Istanbul 34480, Turkey; alicanhatemi@gmail.com

**Keywords:** pediatric, myocarditis, biomarker, systemic immune inflammatory index, systemic inflammatory response index

## Abstract

**Background/Objectives:** Myocarditis is a major cause of morbidity and mortality in children and can lead to long-term heart failure, dilated cardiomyopathy, the need for heart transplantation, or death. New systemic inflammatory indices that combine lymphocyte, neutrophil, and platelet counts have been recently used as strong prognostic markers of some inflammatory diseases and adverse outcomes of neoplasms. This study aimed to investigate the use of new systemic indices as early predictive markers for adverse outcomes in patients with pediatric myocarditis. **Methods:** This study retrospectively examined patients between the ages of >1 month and <18 years who were monitored in our clinic with a diagnosis of myocarditis between 1 January 2022 and 31 December 2024. The cases were divided into two groups: fulminant myocarditis (requiring the use of inotropes or extracorporeal membrane oxygenation due to hemodynamic disturbance) and non-fulminant myocarditis. The systemic inflammatory index values of these groups (calculated in the first 6 h) were compared, and the results were statistically analyzed. **Results:** The study included 122 pediatric myocarditis cases treated during the study period (80 boys; median age: 11 (IQR: 8–14) years). Twenty-six of these patients (21.3%) developed fulminant myocarditis. The median systemic immune-inflammation index (SII) value in the group with fulminant myocarditis was 1300 (IQR: 1000–1600), while this value was 500 (IQR: 350–650) for the non-fulminant group (*p* < 0.05). The median systemic inflammatory response index (SIRI) values were 2.9 (IQR: 2.5–3.2) in the fulminant myocarditis group and 1.5 (IQR: 1.2–1.8) in the non-fulminant group (*p* < 0.05). The cut-off values for fulminant myocarditis were found to be 1050 for the SII, with an AUC value of 0.76 (95% confidence interval: 0.80–0.96; *p* < 0.001), and 1.9 for the SIRI, with an AUC value of 0.64. **Conclusions:** The SII and SIRI may independently predict adverse myocarditis prognoses in children. These new biomarkers are easy to calculate using routine blood parameters.

## 1. Introduction

Myocarditis is an inflammatory disease that affects the myocardium. Although the disease has various etiologies, viruses constitute the most common cause. Childhood myocarditis may be asymptomatic, but can also present with arrhythmia, cardiogenic shock, or sudden death [1]. The incidence of diagnosed myocarditis in children is 1–2 in 100,000 [2,3]. However, asymptomatic cases may remain undetected, and the true frequency of the disease cannot be known for certain.

Although some diagnostic histopathological and clinicopathological criteria have been determined for myocarditis, reliable biomarkers for the early diagnosis of patients at risk of fulminant myocarditis are needed. The use of accurate prognostic biomarkers can help identify high-risk patients early and enhance the likelihood of suitable clinical decisions. Recent studies have shown that new systemic inflammatory indices can be used as promising biomarkers for many diseases, including cancer, coronary artery diseases, and inflammatory diseases [4,5,6].

While the neutrophil-to-lymphocyte ratio (NLR), platelet-to-lymphocyte ratio (PLR), and monocyte-to-lymphocyte ratio (MLR) have been the primary parameters studied in the literature to date, the systemic immune-inflammation index (SII), systemic inflammatory response index (SIRI), and pan-immune inflammation value (PIV) stand out in recent studies. The common characteristics of these parameters are their easy calculation with information from a single complete blood count and their cost-effective nature [4,5,6,7].

Studies evaluating the prognostic value of these inflammatory markers in children diagnosed with acute myocarditis are limited [8]. This study aimed to identify the value of the new inflammatory biomarkers in predicting prognosis and identifying potential adverse outcomes in patients with myocarditis.

## 2. Materials and Methods

### 2.1. Study Design and Patient Selection

This study was retrospectively designed to evaluate patients aged between 1 month and 18 years who underwent treatment for myocarditis in our pediatric cardiology clinic between 1 January 2022 and 31 December 2024. Patients with congenital heart disease, inflammatory or autoimmune diseases, cancer, or incomplete data were excluded.

### 2.2. Definitions

The diagnosis of myocarditis was made based on clinical findings, elevated cardiac biomarkers, ECG and echocardiographic abnormalities, and cardiac MRI findings, as per recent consensus guidelines [3,9]. The Dallas criteria were not systematically applied, as endomyocardial biopsy was not performed in these cases.

Fulminant myocarditis was defined as a clinical picture comprising cardiogenic shock, serious ventricular dysfunction, and/or life-threatening arrhythmia with sudden onset and a rapidly progressing clinical course, requiring inotropic or ventricular support systems [3].

### 2.3. Data Collection

All relevant data, including the demographic characteristics (age, sex, height, body weight, etc.), vital signs, laboratory test results, administered treatments, hospitalization duration, intensive care requirements, intensive care duration, echocardiographic and ECG findings, and cardiac MRI results, were recorded. Laboratory parameters including the white blood cell (WBC) count, absolute neutrophil count (ANC), absolute lymphocyte count (ALC), platelet (PLT) count, procalcitonin (PCT), C-reactive protein (CRP), serum troponin, and pro-brain natriuretic peptide (proBNP) levels were also collected. All patients’ full blood count tests were evaluated in the first 6 h after admission, and their NLRs, PLRs, MLRs, SIIs, SIRIs, and PIVs were calculated. The index calculations were performed as follows: SII = neutrophil count × platelet count/lymphocyte count [10], SIRI = neutrophil count × monocyte count/lymphocyte count [11], and PIV = neutrophil count × platelet count × monocyte count/lymphocyte count [12].

### 2.4. Statistical Analysis

A statistical analysis of the data obtained in this study was performed using IBM SPSS 23.0 for Windows (IBM Corp.). Descriptive statistics including percentages, means, medians, standard deviations (SDs), and interquartile ranges (IQRs) were used to define the population included in the study. To compare the patients with and without acute fulminant myocarditis, chi-square or Fisher exact tests and *t*-tests were used for the categorical and continuous variables, respectively. The Mann–Whitney U test was used for the variables that did not comply with normal distribution. A receiver operating characteristic (ROC) curve analysis was performed, and the ROC curves were plotted to determine the optimal cut-off values of the SII, the SIRI, N-terminal pro-B-type natriuretic peptide (NT-proBNP), and troponin for the prediction of fulminant myocarditis. The area under the curve (AUC) was calculated to predict fulminant myocarditis and compare the performance of each marker. The sensitivity, specificity, positive predictive value (PPV), and negative predictive value (NPV) were calculated for each marker. A Spearman correlation analysis was performed to analyze the correlation between the SII and left ventricular ejection fraction (LVEF). To evaluate the effectiveness of the SII in predicting adverse outcomes in children with myocarditis, the cut-off values were determined by the ROC analysis, and the likelihood ratio was calculated. For all analyses, *p* < 0.05 was considered statistically significant.

### 2.5. Outcome

The primary aim of this study was to determine the value of the inflammatory indices in predicting fulminant myocarditis for various patients and to compare those indices to other cardiac markers used in diagnosing patients with acute myocarditis. The second objective was to evaluate the predictive power of these indices for prognosis.

## 3. Results

**Figure 1:**
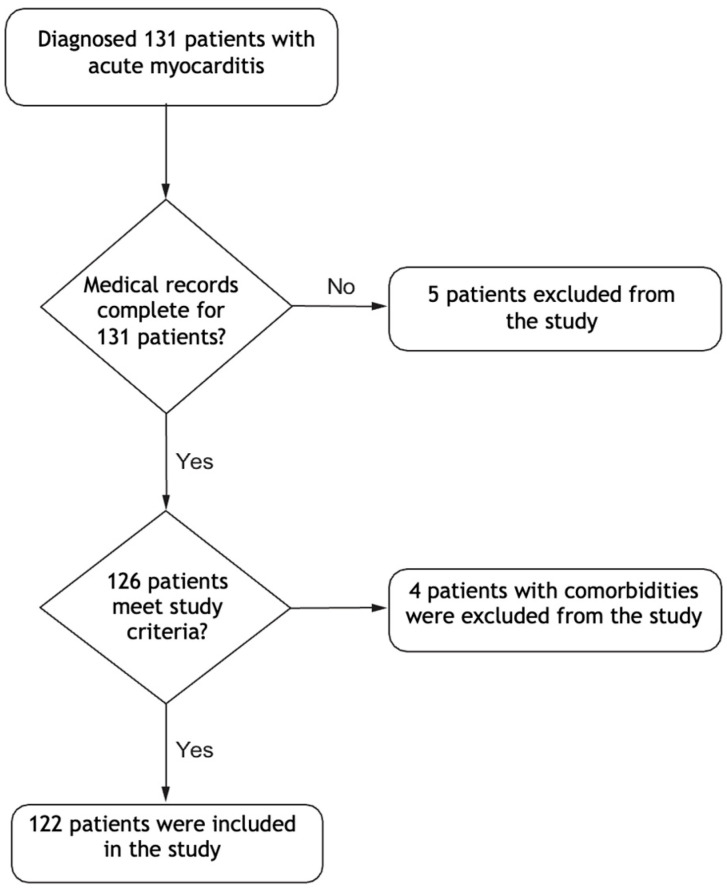
Study flow chart. This figure illustrates the process and sequence of the patient selection, inclusion and exclusion criteria used in this study.

During the study period, 131 patients were followed in our clinic for a diagnosis of acute myocarditis, but only 122 were included in the study. The flowchart of the inclusion process is given in Figure 1. The median age of the included patients was 11 (IQR: 8–14) years, and 65% of the patients were male. No significant difference was found between the fulminant and non-fulminant groups regarding sex. Of the 122 patients, 26 (21%) were diagnosed with fulminant myocarditis. Their median age was 5 (IQR: 3–7) years, while the median age of the patients in the non-fulminant group was 10 (IQR: 8–12) years. Thus, there was a significant difference between the two groups in terms of age (*p* < 0.001). Considering the symptoms of the patients at admission, complaints such as stomachache (50%) and dyspnea (57.7%) were significantly more common in the group with fulminant myocarditis, while chest pain (70%) was significantly more common in the non-fulminant group (*p* < 0.001 for each).

There was a significant difference between the two groups in terms of the basal troponin I values, but no significant difference was found in terms of the peak troponin I values (*p* = 0.035 and *p* = 0.1). The median peak NT-proBNP level in the group with fulminant myocarditis was 32,000 pg/mL (IQR: 25,000–42,000), being significantly higher than that of the non-fulminant myocarditis group (2100 pg/mL [IQR: 100–5600]) (*p* < 0.001). Furthermore, the WBC, ANC, NLR, SII, and SIRI values were significantly higher in the group with fulminant myocarditis (*p* < 0.001 for each). The need for extracorporeal membrane oxygenation (ECMO) support in the fulminant myocarditis group was 26.9% (*n* = 7) and the mortality rate was 15% (*n* = 4), with both values being significantly higher compared to the non-fulminant myocarditis group, which had no cases of ECMO support or mortality (*p* < 0.001). The other laboratory, ECG, echocardiographic, and cardiac MRI data of the patients with fulminant and non-fulminant myocarditis are summarized in Table 1.

**Figure 2:**
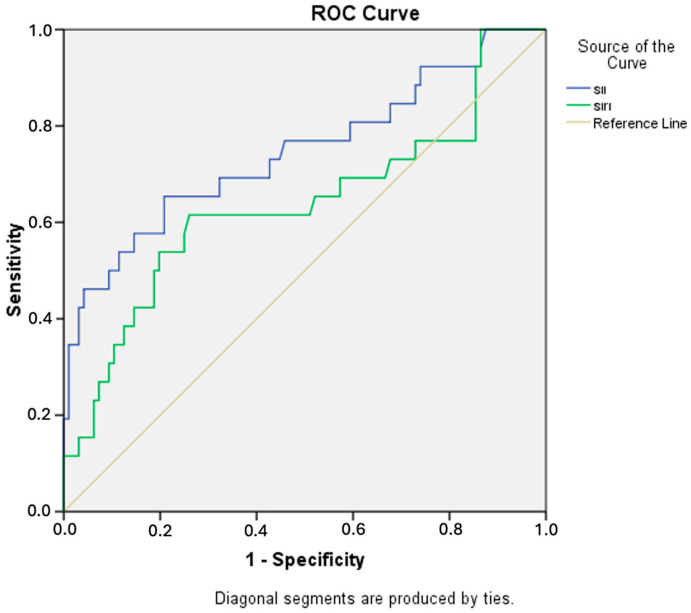
Receiver operating characteristic (ROC) curves for inflammation markers as predictive indicators of fulminant myocarditis. Figure shows ROC curves for SII and SIRI in predicting fulminant myocarditis.

The ROC curves and diagnostic accuracy values of the SII and SIRI in predicting fulminant myocarditis are provided in Figure 2 and Table 2. The SII and SIRI showed acceptable diagnostic power in predicting fulminant myocarditis, with respective AUC values of 0.760 and 0.640. The ROC analysis revealed that the SII had higher diagnostic accuracy, with an AUC of 0.760 (95% confidence interval [CI]: 0.620–0.860). The SII had 80% sensitivity and 90% specificity, with a cut-off value of 1050. On the other hand, the SIRI exhibited 75% sensitivity and 80% specificity, with a cut-off value of 1.9.

This table shows the adjusted odds ratios (aORs) with the 95% confidence intervals (CI) and *p*-values for the predictors of fulminant myocarditis. The predictors analyzed include the SII and SIRI. SII: systemic immune-inflammation index, SIRI: systemic inflammatory response index.

## 4. Discussion

We investigated different variables that affect the course of fulminant myocarditis in pediatric patients diagnosed with acute myocarditis. Younger ages, heart failure symptoms such as respiratory problems and stomachaches at admission, low LVEFs observed by echocardiography, long T2 relaxation periods in cardiac MRIs, high NT-proBNP levels, high WBC and ANC values, and high NLR, SII, and SIRI values were associated with adverse outcomes. Of the new systemic inflammatory indices, the SII and SIRI were found to be independent predictors of fulminant myocarditis. Our study is one of very few studies in the literature to address such findings.

A study conducted by Erbay et al. on young adults diagnosed with myocarditis found the sensitivity and specificity of the SII in diagnosing fulminant myocarditis to be 91% and 83%, respectively [13]. Another study conducted by Yaradılmış et al. on pediatric patients found the sensitivity and specificity of the SII to be 68.8% and 94.4% [8]. Tang et al. reported that there was a strong correlation between high SII levels in critically ill patients with congestive heart failure and adverse short-term outcomes, including 30-day, 90-day, and general hospital mortality rates [14]. Agus et al. showed that the SII was an independent marker of in-hospital mortality in adult patients diagnosed with infective endocarditis [15]. Our study found the SII value to be an independent risk factor for fulminant myocarditis.

No studies on the relationship between the SIRI and fulminant myocarditis were found in the literature; therefore, ours is the first study on this subject. However, in a study conducted by Wang et al. on patients aged 60 or older who were followed due to heart failure, Cox regression analysis results showed that high SIRI values were significantly associated with all-cause mortality. The same study also showed that high SIRI values were associated with hospitalization and duration in intensive care [16]. In their study conducted on patients hospitalized in the intensive care unit due to a diagnosis of sepsis, Xu et al. found a significant relationship between the SIRI values and WBC counts (OR: 0.156; 95% CI: 0.132–0.181), SOFA scores (OR: 0.017; 95% CI: 0.005–0.029), and sepsis prognosis (OR: 1.015; 95% CI: 1.003–1.028) [17]. A Cox analysis of a 20-year cohort study from the United States conducted with 42,875 patients investigated the relationship between the SII and SIRI upon all-cause mortality and cardiovascular mortality risks in adults, and found that individuals with SII levels of >655.56 had higher all-cause mortality risk (hazard ratio [HR]: 1.29; 95% CI: 1.18–1.41) and cardiovascular mortality risk (HR: 1.33; 95% CI: 1.11–1.59) compared to those whose SII levels were <335.36. Furthermore, the all-cause mortality risk (HR: 1.39; 95% CI: 1.26–1.52) and cardiovascular mortality risk (HR: 1.39; 95% CI: 1.14–1.68) of adults with SIRI levels of >1.43 were higher than those with SIRI levels of <0.68 [18].

The PIV is a new parameter, first proposed in 2020 to predict the survival outcomes of patients with metastatic colorectal cancer [12]. There are limited data in the literature on its use in the pediatric age group, and among the very few studies conducted to date, none have investigated the association of the PIV with myocarditis. One study conducted with children with febrile convulsions found the PIV, SII, and SIRI to be significantly higher in the group with febrile convulsions than the healthy control group and the febrile control group [19]. A study investigating the relationship between short-term mortality and PIVs in patients diagnosed with sepsis found that a high PIV was an independent risk factor for 28-day and 90-day all-cause mortality in critical cases of sepsis [20]. We could not find a significant difference in the PIVs between the fulminant myocarditis group and the non-fulminant myocarditis group.

While our study did not find any significant difference between the peak and basal troponin I levels, the peak NT-proBNP levels were significantly higher in the fulminant myocarditis group. Although the troponin levels were increased in the patients with myocarditis and heart failure, studies have shown that there is no relationship between the troponin level and the severity of the disease [21,22]. A study conducted by Zhao et al. to identify the factors associated with the mortality rates of 79 patients diagnosed with fulminant myocarditis found that the BNP levels in the group of surviving patients were significantly higher than those of the deceased patients, and there was no significant difference between the troponin I levels [23]. Another study investigating the clinical and electrocardiographic characteristics of patients diagnosed with fulminant myocarditis categorized the patients into three categories, including those with fulminant myocarditis, those with acute myocarditis, and those with acute pericarditis, and the NT-proBNP levels in the group with fulminant myocarditis were significantly higher than those of the other groups [24]. However, since NT-proBNP is essentially a marker for heart failure, easily calculable markers such as the SII and SIRI would be more valuable in cases of fulminant myocarditis.

## 5. Limitations

This study has some limitations. It was a retrospective study based on single-center data. Another limitation is the small sample size of the group with fulminant myocarditis, which limits the generalizability of the findings. Therefore, their use should be interpreted in conjunction with the clinical and imaging findings. “Although the SII and SIRI have shown potential as early prognostic markers, it is important to note that these indices are not disease-specific and can be elevated in various inflammatory conditions, such as sepsis or infective endocarditis. Therefore, their use should be interpreted in conjunction with the clinical and imaging findings”.

## 6. Conclusions

This study has shown that the new biomarkers of the SII and SIRI, which can be easily calculated based on information from routine blood counts, can help in the early identification of adverse outcomes and predict the course of fulminant myocarditis in children. Prospective studies with larger samples are needed to validate our results.

## Figures and Tables

**Table 1:** Comparative assessment of parameters in fulminant and non-fulminant groups.

Variables	Fulminant (*n* = 26)	Non-Fulminant (*n* = 96)	*p*-Value
Demographic data and symptoms			
Age (years) [median (IQR)]	5 (3–7)	10 (8–12)	<0.001
Gender [*n* (%)]			
Male	17 (65)	63 (66)	0.981
Female	9 (35)	33 (34)	
Symptoms [*n* (%)]			
Chest pain	2 (7.7)	70 (72.9)	<0.001
Fever	3 (11.5)	19 (19.8)	0.403
Palpitation	3 (11.5)	12 (12.5)	0.981
Shortness of breath	15 (57.7)	17 (17.5)	<0.001
Abdominal pain	13 (50)	13 (13.5)	<0.001
URTI	7 (27)	34 (35)	0.488
Laboratory factors (median [IQR])			
Basal troponin I (ng/mL)	138 (90–240)	188 (150–320)	0.035
Peak troponin I (ng/mL)	184 (150–250)	274 (220–350)	0.100
NT-proBNP (pg/mL)	32,000 (25,000–42,000)	2100 (1000–5600)	<0.001
CRP (mg/L)	14 (7–21)	20 (10–40)	0.020
Procalcitonin (ng/mL)	0.5 (0.3–0.9)	0.7 (0.5–1.2)	0.080
WBC (×10^9^/L)	12.7 (10–14.4)	9.4 (8–11)	<0.001
ANC (×10^9^/L)	7.2 (6–8.5)	4.8 (4–6.2)	<0.001
ALC (×10^9^/L)	4.7 (3–6)	2.7 (2.2–4)	0.390
AMC (×10^9^/L)	0.9 (0.6–1.2)	0.7 (0.5–1)	0.010
Platelet (×10^9^/L)	320 (280–360)	280 (2 50–330)	0.470
NLR	2.3 (2–2.7)	1.7 (1.5–1.9)	<0.001
PLR	70 (50–90)	115 (100–130)	0.250
MLR	0.14	0.27	0.060
SII	1300 (1000–1600)	500 (350–650)	<0.001
SIRI	2.9 (2.5–3.2)	1.5 (1.2–1.8)	<0.001
PIV	400 (380–420)	380 (360–400)	0.080
Pathogens identified in patients [*n* (%)]			
Adenovirus	3 (11.5)	5 (5.2)	
Enterovirus/rhinovirus	3 (11.5)	8 (8.3)	
Parainfluenza viruses	2 (7.5)	4 (4.2)	
SARS-Cov-2	2 (7.5)	3 (3.1)	
Other coronaviruses	1 (3.8)	2 (2.1)	
Influenza A-B	-	6 (6.2)	
Others	2 (7.5)	4 (4.2)	
Clinical data [*n* (%)]			<0.001
ECMO	7 (26.9)	0	
In-hospital mortality	4 (15)	0	
Echocardiographic variables			
LVEF (%)	30 (24–35)	45 (40–55)	<0.001
Mitral regurgitation [*n* (%)]			<0.001
Mild	2 (7.5)	8 (8.3)	
Mild to moderate	4 (15)	2 (2.1)	
Moderate	6 (22.5)	1 (1.1)	
Moderate to severe	8 (30)	-	
Severe	6 (22.5)	-	
Pericardial effusion [*n* (%)]	4 (15.3)	12 (12.5)	0.875
ECG variables [*n* (%)]			<0.001
No findings	-	24 (25)	
Sinus tachycardia	13 (50)	16 (16.6)	
ST elevation	8 (30)	12 (12.5)	
ST depression	3 (11.5)	8 (8.3)	
Ventricular extrasystoles	8 (30)	2 (2.1)	
CMR parameters (median [IQR])			
LGE (%)	4 (2–6)	3 (2–4)	0.650
T1 relaxation time (ms)	1200 (1000–1400)	1000 (800–1100)	0.003
T2 relaxation time (ms)	68 (60–76)	48 (40–56)	0.001
ECV fraction (%)	15 (10–25)	18 (10–30)	0.890
Pericardial effusion [*n* (%)]	5 (19.2)	14 (14.5)	0.420

URTI: Upper respiratory tract infection, NT-proBNP: N-terminal pro-brain natriuretic peptide, CRP: C-reactive protein, WBC: White blood cell count, ANC: Absolute neutrophil count, ALC: Absolute lymphocyte count, AMC: Absolute monocyte count, NLR: Neutrophil-to-lymphocyte ratio, PLR: Platelet-to-lymphocyte ratio, MLR: Monocyte-to-lymphocyte ratio, SII: Systemic immune-inflammation index, SIRI: Systemic inflammatory response index, PIV: Pan-immune inflammation value, ECMO: Extracorporeal membrane oxygenation, LVEF: Left ventricle ejection volume, ECG: Electrocardiogram, CMR: Cardiac magnetic resonance imaging, LGE: Late gadolinium enhancement, ECV: Extracellular volume.

**Table 2:** Logistic analysis for predictors of fulminant myocarditis.

Parameters	AUC	Cut-Off	Sensitivity %	Specificity %	95% CI	*p*-Value
SII	0.760	1050	80	90	0.620–0.860	0.001
SIRI	0.640	1.9	75	80	0.500–0.770	0.03

## Data Availability

The original contributions presented in this study are included in the article. Further inquiries can be directed to the corresponding author.

## References

[1] Kim J., Cho M.-J. (2020). Acute myocarditis in children: A 10-year nationwide study (2007–2016) based on the Health Insurance Review and Assessment Service Database in Korea. Korean Circ. J..

[2] Vasudeva R., Bhatt P., Lilje C., Desai P., Amponsah J., Umscheid J., Parmar N., Bhatt N., Adupa R., Pagad S. (2021). Trends in acute myocarditis related pediatric hospitalizations in the United States, 2007–2016. Am. J. Cardiol..

[3] Drazner M.H., Bozkurt B., Cooper L.T., Aggarwal N.R., Basso C., Bhave N.M., Caforio A.L., Ferreira V.M., Heidecker B. (2025). 2024 ACC Expert Consensus Decision Pathway on Strategies and Criteria for the Diagnosis and Management of Myocarditis: A Report of the American College of Cardiology Solution Set Oversight Committee. J. Am. Coll. Cardiol..

[4] Dincer A., Sezer S. (2022). Systemic immune inflammation index as a reliable disease activity marker in psoriatic arthritis. JCPSP-J. Coll. Physicians Surg. Pak..

[5] Zhang Y., Xing Z., Zhou K., Jiang S. (2021). The predictive role of systemic inflammation response index (SIRI) in the prognosis of stroke patients. Clin. Interv. Aging.

[6] Guven D.C., Sahin T.K., Erul E., Kilickap S., Gambichler T., Aksoy S. (2022). The association between the pan-immune-inflammation value and cancer prognosis: A systematic review and meta-analysis. Cancers.

[7] Yükcü B., Arslan H.F. (2024). New systemic inflammatory indices as predictors of ascending aortic dilation in children with bicuspid aortic valve: A retrospective cross-sectional study. Medicine.

[8] Yaradilmiş R.M., Güneylioğlu M.M., Öztürk B., Göktuğ A., Aydın O., Güngör A., Bodur İ., Kaya Ö., Örün U.A., Karacan C.D. (2023). A novel marker for predicting fulminant myocarditis: Systemic immune–inflammation index. Pediatr. Cardiol..

[9] Caforio A.L., Pankuweit S., Arbustini E., Basso C., Gimeno-Blanes J., Felix S.B., Fu M., Heliö T., Heymans S., Jahns R. (2013). Current state of knowledge on aetiology, diagnosis, management, and therapy of myocarditis: A position statement of the European Society of Cardiology Working Group on Myocardial and Pericardial Diseases. Eur. Heart J..

[10] Hu B., Yang X.-R., Xu Y., Sun Y.-F., Sun C., Guo W., Zhang X., Wang W.-M., Qiu S.-J., Zhou J. (2014). Systemic immune-inflammation index predicts prognosis of patients after curative resection for hepatocellular carcinoma. Clin. Cancer Res..

[11] Qi Q., Zhuang L., Shen Y., Geng Y., Yu S., Chen H., Liu L., Meng Z., Wang P., Chen Z. (2016). A novel systemic inflammation response index (SIRI) for predicting the survival of patients with pancreatic cancer after chemotherapy. Cancer.

[12] Fucà G., Guarini V., Antoniotti C., Morano F., Moretto R., Corallo S., Marmorino F., Lonardi S., Rimassa L., Sartore-Bianchi A. (2020). The Pan-Immune-Inflammation Value is a new prognostic biomarker in metastatic colorectal cancer: Results from a pooled-analysis of the Valentino and TRIBE first-line trials. Br. J. Cancer.

[13] Erbay I., Kokturk U., Eris Gudul N., Avci A. (2024). Prognostic role of systemic immune-inflammation index versus other cardiac markers in acute myocarditis in young adults. Biomark. Med..

[14] Tang Y., Zeng X., Feng Y., Chen Q., Liu Z., Luo H., Zha L., Yu Z. (2021). Association of systemic immune-inflammation index with short-term mortality of congestive heart failure: A retrospective cohort study. Front. Cardiovasc. Med..

[15] Agus H.Z., Kahraman S., Arslan C., Yildirim C., Erturk M., Kalkan A.K., Yildiz M. (2020). Systemic immune-inflammation index predicts mortality in infective endocarditis. J. Saudi Heart Assoc..

[16] Wang X., Ni Q., Wang J., Wu S., Chen P., Xing D. (2022). Systemic inflammation response index is a promising prognostic marker in elderly patients with heart failure: A retrospective cohort study. Front. Cardiovasc. Med..

[17] Xu T., Song S., Zhu K., Yang Y., Wu C., Wang N., Lu S. (2025). Systemic inflammatory response index improves prognostic predictive value in intensive care unit patients with sepsis. Sci. Rep..

[18] Xia Y., Xia C., Wu L., Li Z., Li H., Zhang J. (2023). Systemic immune inflammation index (SII), system inflammation response index (SIRI) and risk of all-cause mortality and cardiovascular mortality: A 20-year follow-up cohort study of 42,875 US adults. J. Clin. Med..

[19] Söğütlü Y., Altaş U. (2024). Predictive Value of Neutrophil–Lymphocyte Ratio and Other Inflammation Indices in Febrile Seizures in Children. J. Clin. Med..

[20] Xu H.-B., Xu Y.-H., He Y., Lin X.-H., Suo Z., Shu H., Zhang H. (2024). Association between admission pan-immune-inflammation value and short-term mortality in septic patients: A retrospective cohort study. Sci. Rep..

[21] Soongswang J., Durongpisitkul K., Nana A., Laohaprasittiporn D., Kangkagate C., Punlee K., Limpimwong N. (2005). Cardiac troponin T: A marker in the diagnosis of acute myocarditis in children. Pediatr. Cardiol..

[22] Butto A., Rossano J.W., Nandi D., Ravishankar C., Lin K.Y., O’Connor M.J., Shaddy R.E., Shamszad P. (2018). Elevated troponin in the first 72 h of hospitalization for pediatric viral myocarditis is associated with ECMO: An analysis of the PHIS+ database. Pediatr. Cardiol..

[23] Zhao Y., Da M., Yang X., Xu Y., Qi J. (2024). A retrospective analysis of clinical characteristics and outcomes of pediatric fulminant myocarditis. BMC Pediatr..

[24] Itoh T., Kobayashi T., Oshikiri Y., Arakawa Y., Satoh M., Morino Y. (2022). Clinical and electrocardiographic characteristics in patients with fulminant myocarditis. J. Arrhythmia.

